# Correlations between microsatellite instability and the biological behaviour of tumours

**DOI:** 10.1007/s00432-019-03053-4

**Published:** 2019-10-15

**Authors:** Guang Yang, Ru-yi Zheng, Zai-shun Jin

**Affiliations:** 1 Department of Pathology, Okayama University Graduate School of Medicine, Dentistry and Pharmaceutical Sciences, Okayama, Japan; 2 Medical Imaging Center, The Mine Hospital of Xu Zhou, Xuzhou, Jiangsu China; 3 Mudanjiang Medical University, Mudanjiang, Heilongjiang 157000 China

**Keywords:** Carcinoma, Microsatellite instability, Frameshift peptide, Prognosis, Targeted therapies

## Abstract

**Purpose:**

Microsatellites are widely distributed repetitive DNA motifs, accounting for approximately 3% of the genome. Due to mismatch repair system deficiency, insertion or deletion of repetitive units often occurs, leading to microsatellite instability. In this review, we aimed to explore the relationship between MSI and biological behaviour of colorectal carcinoma, gastric carcinoma, lymphoma/leukaemia and endometrial carcinoma, as well as the application of frameshift peptide vaccines in cancer therapy.

**Methods:**

The relevant literature from PubMed and Baidu Xueshu were reviewed in this article. The ClinicalTrials.gov database was searched for clinical trials related to the specific topic.

**Results:**

Microsatellite instability is divided into three subtypes: high-level, low-level microsatellite instability, and stable microsatellites. The majority of tumour patients with high-level microsatellite instability often show a better efficacy and prognosis than those with low-level microsatellite instability or stable microsatellites. In coding regions, especially for genes involved in tumourigenesis, microsatellite instability often results in inactivation of proteins and contributes to tumourigenesis. Moreover, the occurrence of microsatellite instability in coding regions can also cause the generation of frameshift peptides that are thought to be unknown and novel to the individual immune system. Thus, these frameshift peptides have the potential to be biomarkers to raise tumour-specific immune responses.

**Conclusion:**

MSI has the potential to become a key predictor for evaluating the degree of malignancy, efficacy and prognosis of tumours. Clinically, MSI patterns will provide more valuable information for clinicians to create optimal individualized treatment strategies based on frameshift peptides vaccines.

## Introduction

Microsatellites are repetitive DNA motifs that are widely distributed within the genome and closely linked to many important genes (Cullis 2002). Due to mutation or epigenetic changes of DNA mismatch repair (MMR) genes, normal function of the DNA MMR system is destroyed, and the number of microsatellite base pairs experiences alteration known as microsatellite instability (MSI).

Many studies have shown that MSI plays an important role in the pathogenesis of malignant tumours and is closely related to the occurrence, progression, and prognosis of many malignancies. The majority of studies have revealed that patients with high levels of MSI (MSI-H) exhibit a better anti-tumour immune response, the ability to inhibit tumour cell growth, and improved prognosis compared to those with low levels of MSI (MSI-L) or who are microsatellite stable (MSS) (Choi et al. 2014; Kim et al. 2016; Marrelli et al. 2016; Mohan et al. 2016; Smyrk et al. 2001). Therefore, MSI has the potential to become a key predictor for evaluating the degree of malignancy, efficacy, and prognosis of tumours. Furthermore, MSI patterns will provide more valuable information for clinicians to create individualized treatment strategies.

MSI occurring in the coding regions of genes is known as coding MSI (cMSI). cMSI can result in inactivation of proteins crucial for suppressing tumourigenesis and production of frameshift peptides (FSPs). Theoretically, these FSPs are novel, unknown, and tumour-specific to the individual immune system, because they are only produced by clonal tumour cells. At present, many FSP vaccines have been artificially synthesized and successfully applied in basic research and clinical therapy. Thus, it is projected that FSPs will become targets of immune therapy, and they are of great significance for preventing and treating malignancies in a targeted way.

Currently, MSI has been frequently observed in various malignancies and has become a new research hotspot. Thus, we will next present a review of the relationship between MSI and biological behaviour of colorectal carcinoma (CRC), gastric carcinoma (GC), lymphoma/leukaemia (L/L), and endometrial carcinoma (EC), as well as the application of FSP vaccines in cancer therapy.

## Microsatellites and MSI

Microsatellites are simple sequence repeats (SSRs) that are widely distributed within the biological genome. These repetitive units consist of 1–6 nucleotides (Vaksman and Garner 2015). Although microsatellites are widely distributed within the genome, their distribution patterns are not random. They are distributed far more in non-coding regions of genes than in coding regions in eukaryotes. Furthermore, microsatellites are considered to play an important role in the formation and reorganization of chromosomal structures, which can affect gene replication and expression (Chistiakov et al. 2006).

MSI is defined as a change of microsatellite length caused by insertion or deletion of a repetitive unit, leading to the appearance of new microsatellite alleles. According to the mutant number of microsatellite sites, MSI is categorized as three different subtypes, including high levels of MSI (MSI-H), low levels of MSI (MSI-L), and stable microsatellite (MSS) (Boland et al. 1998). Initially, there was no uniform standard for MSI detection in cancer, impeding relevant microsatellite studies. In 1997, the American National Cancer Institute (NCI) recommended five microsatellite loci (BAT 25, BAT 26, D2S123, D5S346, and D17S250), among which BAT 25 and BAT 26 are mononucleotide repeats; D5S346, D2S123, and D17S250 are dinucleotide repeats, as preferred biomarkers used for MSI research. As described, MSI-H indicates greater than or equal to two microsatellite loci exhibiting mutations; MSI-L indicates only one locus is mutated; and MSS indicates an absence of mutations (Boland et al. 1998). Compared with MSS, MSI status is a feasible predictor for the choice of treatment method in MSI-H and MSI-L tumours (Caliman et al. 2012). In addition, MSI is a proven indicator of efficacy, prognosis, and recurrence in CRC, GC, and other cancers, and is of great significance for studying the pathogenesis of carcinomas (Caliman et al. 2012; Gemayel et al. 2010; Kang et al. 2015).

## Molecular mechanism of MSI occurrence

The mismatch repair (MMR) system is responsible for recognizing and repairing mismatched bases during DNA replication, especially in repetitive DNA sequences, such as microsatellites (Jiricny 2013). DNA MMR deficiency is one of the most important mechanisms leading to underlying genomic instability, chromosomal instability, and MSI. It has been found that DNA MMR-related proteins consist at least of seven types, including h-MLH1, h-MLH3, hMSH2, h-MSH3, h-MSH6, h-PMS1, and h-PMS2. Functional heterodimers are formed by specific combinations between MMR-related proteins to recognize mismatched base pairs, including insertion or deletion of small nucleotides (1–4 base pairs) during DNA replication (Velho et al. 2014). DNA MMR is a highly conserved intracellular process involving a variety of proteins. During the process of DNA replication, due to gene recombination or physical and chemical damage to bases, the DNA MMR system is activated to identify and repair the errors. However, when DNA MMR-related genes experience mutation or epigenetic changes, the genes lose their ability to synthesize MMR-related proteins, which further results in DNA MMR deficiency and MSI occurrence (Choi et al. 2014; Lynch and de la Chapelle 2003).

## The correlation between MSI and the biological behaviour of malignancies

### MSI and colorectal carcinoma

The MSI phenotype that is the most closely associated with CRC was first identified in CRC, and relevant studies are relatively mature. In addition, a perfect criterion has also been established to evaluate MSI status in CRC.

CRC is a major health problem, causing 70,000 deaths every year worldwide. It is the second and third most common malignant tumour in men and women, respectively (Chang et al. 2017). Fifteen percent of CRC patients show DNA MMR deficiency with MSI-H (Lynch and de la Chapelle 2003). Among MSI CRC patients, the majority appear to exhibit DNA MMR gene *MLH1* or *MSH2* mutation (Bonadona et al. 2011) or occurrence of hyper-methylation of the *MLH1* promoter (Torre et al. 2015). To explore the correlation between MSI and the biological behaviour of CRC, many studies collected and analyzed a large number of cases. Wade S. Samowitz et al. (2015) revealed that CRC patients with MSI-H exhibited reduced invasive capability compared to those with MSI-L, which was consistent with results from Lynch HT (Lynch et al. 2015). Mohan et al. (2016) was engaged in a study involving 1250 CRC patients and found that stage I/II CRC patients with MSI-H had a lower risk of lymph node or distant metastasis with significantly improved disease-free survival (DFS). However, Kim et al. (2016) performed a study comprising 2940 CRC patients with stage I–III CRC, showing that patients with MSI-H had better clinical prognosis, but were often accompanied by local recurrence and peritoneal metastasis. This result was slightly different from the previous work by Mohan, which may be primarily attributed to there being a significant difference in the number of patient samples or microsatellite markers used in the studies.

The reason for CRC patients with MSI-H exhibiting better prognosis was explored by investigators who revealed that this phenomenon may be due to a strong anti-tumour immune response elicited by the patients themselves. Smyrk et al. (2001) performed MSI analysis of 138 CRC patients, demonstrating that parents with MSI-H exhibited high-density infiltrating lymphocytes in their lesions. Therefore, the number of tumour-infiltrating lymphocytes may be helpful for predicting MSI subtypes. In addition, the function of tumour-infiltrating lymphocytes in CRC patients was examined in detail by Badalamenti et al. (2018). They found that these infiltrating lymphocytes primarily consist of cytotoxic T lymphocytes (CTL) that can raise a more highly specific anti-tumour immune response, indicating that high-density tumour-infiltrating lymphocytes have the ability to inhibit invasion and infiltration of MSI-H CRC and improve autologous anti-tumour immune response to obtain improved efficacy and prognosis.

### MSI and gastric carcinoma

GC remains a considerable health burden throughout the world and is one of the most common malignant tumours and the third cancer-related cause of death (Charalampakis et al. 2018; Torre et al. 2015). Although its morbidity and mortality have decreased slightly in the past 30 years, GC still threatens health around the world, especially in China, Japan, and some other southeastern countries in Asia, and the 5-year survival rate of advanced GC patients is not ideal.

MSI is considered to be closely related to tumourigenesis. Therefore, correlations between MSI and the occurrence, prognosis, and chemosensitivity of GC have been studied by many researchers. The primary mechanism of MSI occurrence in GC is distinct from that in CRC. In CRC, the major reason for MSI is DNA MMR gene *MLH1* or *MSH2* mutation. However, while an *MLH1* or *MSH2* mutation is relatively rare in MSI-H GC, methylation of *MLH1* promoter is frequently observed (Ottini et al. 2006).

Some studies (Li et al. 2015; Sugimoto et al. 2016) have reported that MSI exists in precancerous lesions and MSI detection rate appears to be a growing tendency during the progression from precancerous lesions to GC. Thus, MSI may represent a potential molecular indicator for early diagnosis and prophylaxis of GC and is of great significance in precancerous lesions.

At present, there is much controversy concerning the relationship between MSI and prognosis of GC. Choi et al. (2014) reported that MSI-H GC has different biological characteristics compared to MSI-L and MSS GC. In detail, the pooled hazard ratio (HR) for overall survival of MSI-H vs non-MSI-H was 0.72 (95% CI: 0.59–0.88, *p* = 0.001) in a random-effects model, indicating that MSI-H GC patients usually have better prognosis than do MSI-L or MSS patients. Furthermore, Zhu et al. (2015) conducted a meta-analysis on the correlation between MSI and GC, obtaining the same conclusion as above. In addition, Marrelli et al. (2016) performed a relevant analysis between intestinal non-cardiac type GC and MSI, finding that the 5-year survival rate of intestinal non-cardiac type GC with MSI-H was significantly higher than in MSS GC (67.6% vs. 35%, *p* < 0.001). Therefore, it seems that the MSI subtype is a potential predictor of long-term prognosis in intestinal non-cardiac type GC patients.

However, it is believed that the prognosis of GC patients assessed only by MSI status is very difficult to use as a sole predictor, because prognosis may also be affected by other factors, such as patient age, the stage and grade of GC, and so forth. According to Polom’s study (Polom et al. 2017), the prognosis of GC patients has a much more significant correlation with age than MSI status. Specifically, in patients greater than 65 years old, the prognosis of MSI GC patients was better, but in younger GC patients (< 65 years old), there was no statistical significance between prognosis and MSI subtypes. An et al. (2012) obtained a very different result, indicating that DFS curves show no significant differences between MSS/MSI-L and MSI-H GC patients at any stage (I, II, III and IV). They contend that MSI status does not significantly affect patient DFS after receiving 5-fluorouracil-based chemotherapy.

Therefore, the prognosis of GC patients is influenced by many factors, including MSI patterns, stage, grade, age, and chemotherapy treatment. MSI subtypes can be used as an auxiliary reference index combined with other factors to evaluate the prognosis of GC patients, but they cannot be used as an absolute or as the sole index to assess prognosis. Currently, a clear correlation between MSI and prognosis in GC requires additional study.

In addition, the detailed molecular mechanism that MSI patterns affect the prognosis of GC patients was systematically researched among 295 patients by Hang et al. (2018). The results indicated that the effect of MSI on the prognosis of GC patients may be mediated by ten important pathways, including measles, antigen processing and presentation, rheumatoid arthritis, phagosome, systemic lupus erythaematosus, herpes simplex infection, inflammatory bowel disease, tuberculosis, type I diabetes mellitus, and toxoplasmosis pathways. Among them, both inflammation- and immune-related antigen processing and presentation, as well as inflammatory bowel disease pathways, were thought to be the most important mechanisms involved in the prognosis of MSI + GC patients.

### MSI and lymphoma/leukaemia

Recently, MSI detection technology has been widely used for evaluation of haematological malignancies and has been reported in many studies. MSI phenotypes were first observed in CRC and some studies on MSI are relatively mature. A perfect set of standards has been established to judge MSI status in CRC (Yamamoto and Imai 2015). However, for haematological malignancies, there are no unified criteria for the determination of the MSI status. The MSI status in the same haematological tumour cell line is not consistent among different studies. Therefore, it is necessary to identify a panel of highly sensitive and specific MSI biomarkers in haematological malignancies.

In 2016, Miyashita et al. (2017) first used five microsatellite markers (D2S123, D5S107, D10S197, D11S904, and D13S175) to detect MSI in tumours and normal tissues from 20 adult T-cell leukaemia/lymphoma (ATLL) patients. Expression of MMR proteins was also evaluated by immunohistochemistry (IHC). They found that 4 of 20 ATLL patients exhibited MSI. Interestingly, occurrence of MSI was often accompanied by loss or decrease of DNA MMR proteins (Arulananda et al. 2018). However, in this study, because methylation of the MMR gene promoter is very rare in ATLL tumours, expression of MMR proteins MSH2 and MLH1 did not significantly decrease as assessed by IHC, which was consistent with previous studies (Matsushita et al. 2005). In my opinion, perhaps, it was because in ATLL, the occurrence of MSI+ involves other molecular abnormalities that have nothing to do with the deficiency of MMR proteins. In addition, they further analyzed clinical results of these patients and found that patient survival was significantly worse in the MSI+ group (*p* = 0.041), exhibiting obvious resistance to chemotherapeutic adjuvant treatment. All MSI+ patients succumbed to the disease within 4 years. In contrast, the results of MSS patients were significantly better than those with MSI+ . Intriguingly, the results of this study are contrary to those of CRC patients (Koi et al. 2018). The primary reason for this discrepancy is because MSI+ ATLL had characteristics of resistance to chemotherapeutic drugs, so chemotherapy was not effective in these patients. However, at present, the detailed mechanism related to resistance to chemotherapeutic drugs in MSI+ ATLL patients is still not well understood.

In 2017, 1394 acute myeloid leukaemia (AML) patients were assessed for MSI based on next-generation sequencing (NGS) by Christopher Walker et al. (2017). Currently, this is the largest detected population for AML. However, none of them appeared to be MSI+, so it was believed that the MSI phenomenon is extremely rare or nonexistent in adult de novo AML patients at diagnosis. This result was in stark contrast from previous studies, showing that t-AML might be more prone to MSI (Herzog et al. 2005). The primary reason for the disparity produced between these studies might be due to individual differences between the two data sets, as well as differences in MSI detection methods and MSI marker selection.

However, from our point of view, we favour research results achieved by Christopher, because NGS techniques not only allow greater numbers of microsatellites to be assessed compared to conventional MSI screening methods but also possess high detection efficiency (Salipante et al. 2014). In past studies, the techniques frequently used for MSI detection were gel electrophoresis. In this method, migration of DNA fragments is error-prone and sometimes misread, which leads to potential false positives.

In addition, Niv et al. (2005) examined the stability of microsatellites associated with the development of chronic B lymphocyte leukaemia (CLL) or DNA MMR deficiency. It was found that 4 of 27 samples showed positive replication errors compared to normal tissues. Compared to stage A or B CLL, a higher proportion of stage C patients exhibited positive replication errors. Moreover, compared to patients with CLL alone, those complicated by other malignancies showed a higher proportion of positive replication errors. Thus, MSI may play a more dominant role in the pathogenesis and progression of CLL.

In different types of lymphoma/leukaemia (L/L), MSI phenotype and their effects on disease are different as well. Therefore, a clear correlation between MSI and biological behaviour of L/L requires further research.

### MSI and endometrial carcinoma

EC is one of the most common MSI tumours among sporadic malignancies. Similar to CRC, MSI-H is considered to be a prominent feature of EC (Yeramian et al. 2013). Previously, Eto T established a unique high-resolution fluorescence microsatellite analysis (HRFMA) system to accurately and quantitatively analyze PCR products of tumour samples (Eto et al. 2016). Some aspects of MSI that had not been recognized in human malignancies were first revealed by Eto T using this system. In particular, they found two new MSI patterns, type A (microsatellite length change ≤ 6 bp) and type B (microsatellite length change ≥ 8 bp). Among 94 EC cases, 38 were observed to have significant microsatellite motif changes. Compared to the MSI-H/L subtype, the type A/B modality of MSI is more correlated with clinicopathological characteristics and the molecular background of tumours. More importantly, type B MSI is also associated with hereditary non-polyposis colorectal carcinoma-related (HNPCC-related) cancer. In addition, proto-oncogenes associated with MSI were also detected. In MSI+ tumours, the mutation frequency of the *KRAS* gene is controversial. Initially, mutation of this gene was thought to be uncommon in MSI+ tumours (Samowitz et al. 2001). However, they found that *KRAS* gene mutations were closely related to type A MSI, which may suggest that the A/B classification is more biologically relevant.

In recent years, the mechanism of immune escape by tumour cells has become a popular research topic. The PD-1/PD-L1 pathway is generally considered to be one of the most important mechanisms of immune escape. PD-L1, showing widespread expression on the surface of tumour cells, is a negative regulator of T-cell immune function. Therefore, blockage of PD-1/PD-L1 pathway can lead to enhanced activation of anti-tumour immune response (Llosa et al. 2015). The anti-PD-1/PD-L1 antibody has been widely used in the treatment of cancers and has achieved a good efficacy, but this does not mean that the antibody has a good therapeutic effect in all tumour patients (Nghiem et al. 2016). Recently, the relationship between MSI status and PD-L1 expression has been revealed. Many studies have found a positive correlation between MSI status and PD-L1 expression. In MSI-H cancer, a large number of molecules related to immune escape were expressed in tumour-infiltrating lymphocytes and tumour cells, e.g., PD-1 and PD-L1 (Llosa et al. 2015; Ma et al. 2016). In contrast, MSS tumour cells and tumour-infiltrating lymphocytes showed very little immune checkpoints (Kelderman et al. 2015; Llosa et al. 2015; Thompson et al. 2017). In a phase II prospective clinical trial, pembrolizumab, an anti-PD-1 antibody, was used to treat a group of refractory metastatic patients with MMRD colorectal cancer, MMR-proficient colon cancer, or MMRD non-colorectal cancer (Asaoka et al. 2015). Results showed that MSI status was found to be an important predictor of overall response rate (ORR)—40% of ORR in MMRD colorectal cancer, 71% in MMRD non-colorectal cancer, and 0% in MMR-proficient colorectal cancer and progression-free survival rate (78%, 67%, and 11% in each subgroup, respectively). It was obvious that the prognosis of patients with MSI was better than that of patients with MSS after receiving an immune-checkpoint inhibitor therapy. Yamashita et al. (2018) researched 149 EC patients, finding in the MSI+ EC group that expressions of CD8 and PD-1 on the surface of infiltrating lymphocytes and PD-L1 on the surface of tumour cells were significantly higher than in MSS EC. This indicates that MSI+ may be a feasible biomarker for good response to anti-PD-1/PD-L1 immunotherapy. Therefore, the data suggest that anti-PD-1/PD-L1 antibody will be more effective at blocking the PD-1/PD-L1 pathway and allowing a stronger activation of anti-tumour immune response in MSI+ cancers than in those with MSS. Therefore, it is believed that MSI has the potential to become an important predictor for treatment of immune-checkpoint inhibitors. These results provide us with insight that using MSI as an additional predictive biomarker of response to immune therapy may improve therapeutic scenario clinically.

### MSI in coding regions and frameshift peptides

**Fig. 1:**
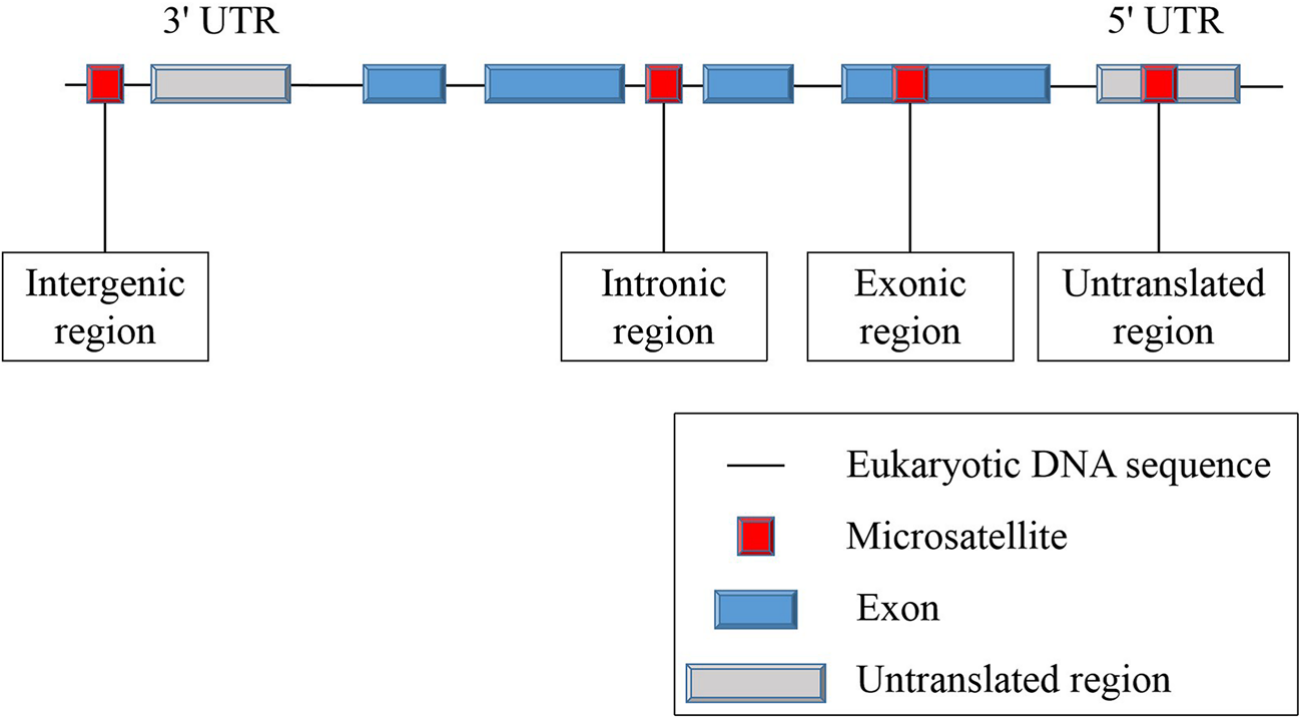
Distribution of microsatellites in the human genome

Microsatellites are primarily located in non-transcriptional and non-translational regions of genes. Most of the length changes in microsatellites between genes are functionally neutral (Sammalkorpi et al. 2007). However, gene-related sequences also contain microsatellite sites in introns, exons, and non-coding regions (Fig. 1). When located in protein-coding regions, microsatellites are known as coding microsatellites (cMS). cMS mutations of some special genes, such as *TGFβRII* and *Bax* (Rampino et al. 1997; Wang et al. 1995), seem to trigger tumourigenesis in MMR-deficient cells, leading to functional inactivation of genes involved in many pathways related to crucial characteristics of cancers (Woerner et al. 2006). Therefore, cMS mutations, also known as coding MSI (cMSI), are considered to be a key event in the development of MSI cancer (Alhopuro et al. 2012; Woerner et al. 2006).

**Fig. 2:**
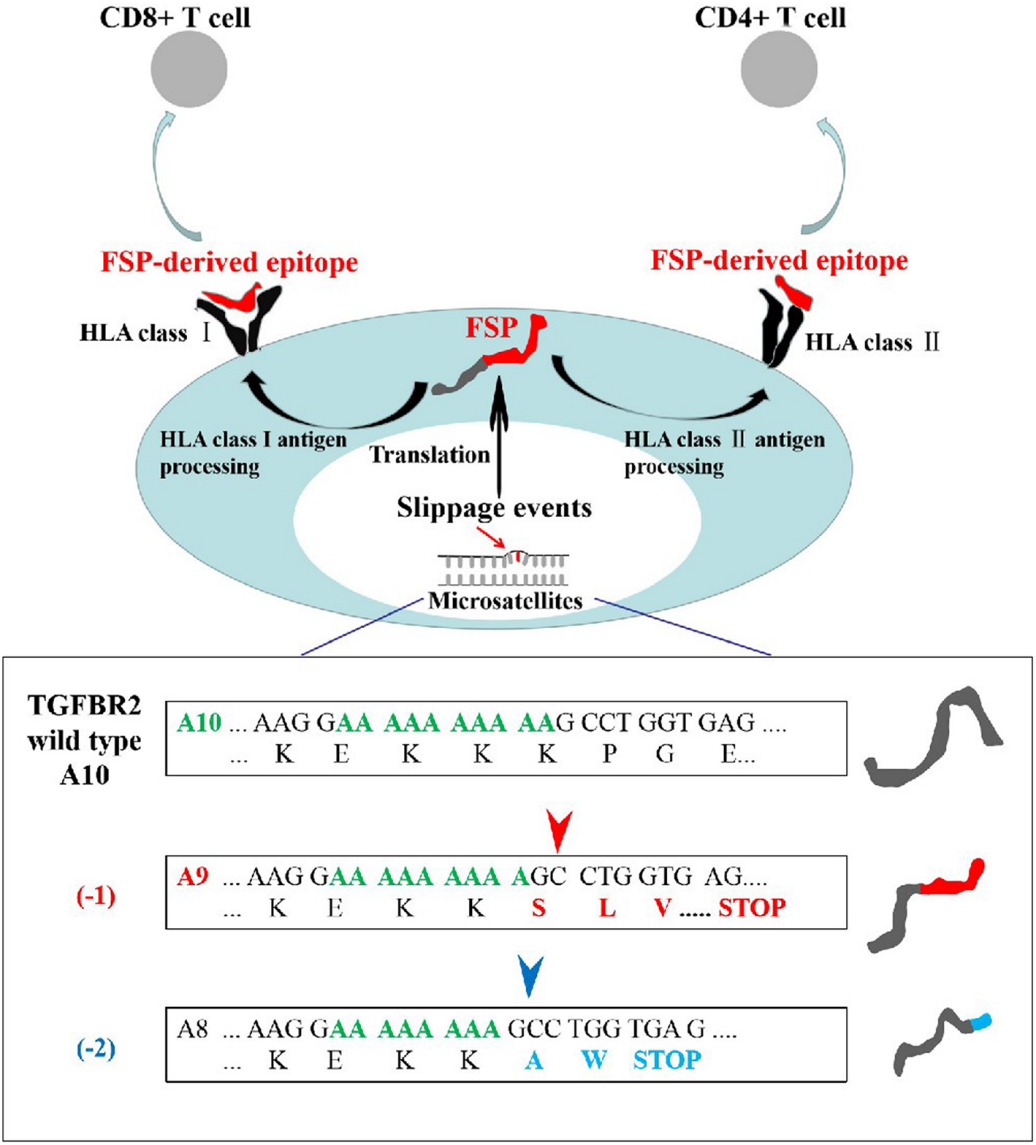
Tumour–immune cell interactions in microsatellite-instable (MSI) cancer (e.g., *TGFβIIR* gene)

cMSI not only promotes tumourigenesis by abrogating the function of proteins involved in tumour suppressor signals, but also induces the generation of novel peptide antigens (truncated proteins) due to frameshifts, known as frameshift peptides (FSPs). Theoretically, these FSPs are true tumour-specific antigens, because they are only produced by clonal cancer cells and then presented by human leukocyte antigen (HLA) on the surface of cancer cells (Fig. 2). FSPs are completely unknown and novel to the individual immune system, making them ideal targets for specific immune responses mediated by cytotoxic T lymphocytes (CTL) to recognize and eradicate existing cancer cells or to inhibit excessive growth of tumour lesions.

As for peptide vaccine therapy, Maletzki et al. (2013) were engaged in FSP research for coding microsatellite-containing genes in lymphoma/leukaemia (L/L) cell lines. Five genes (*TGFβIIR, OGT, FTO, Casp5,* and *MSH3*) from 33 coding microsatellite- containing genes were identified through a screen as target genes used for the following FSP research. By enzyme-linked immunospot (ELISpot) assay used to detect specific recognition of CTLs, the researchers found that FSP-activated and human leukocyte antigen-A2 (HLA-A2)-restricted T cells specifically recognized MSI+ L/L cells endogenously expressing the same FSPs. Moreover, by chromium release assay, the specific lysis ability of CTLs was also identified in L/L cell lines expressing Caspase 5(-1) and MSH3(-1). In vivo, peptide vaccines have also been proven to have a characterization of compelling anti-tumour efficacy. About 50% of human melanomas are strongly associated with *BRAF* mutations, which renders tumour patients with this type of melanoma usually have high immunosuppression and resistance to vaccine treatment (Junttila and de Sauvage 2013). Therefore, based on *BRAF* mutation status, BRAF^V600E^ peptide was synthesized artificially as an immunotherapy drug for the treatment of melanoma in C57BL6 mice in a study conducted by Liu et al. (2018). By IFN-γ production assay and CTL assay, BRAF-specific immune response was illustrated and a large amount of CTL infiltration was observed within the tumour microenvironment. Compared with the control group, a robust, antigen-specific cytotoxic T-cell response and potent tumour growth inhibition were elicited by BRAF peptide vaccine in a murine *BRAF*-mutant melanoma model. Thus, BRAF-based peptide vaccine is a promising strategy for the BRAF-mutant melanoma therapy.

Clinically, Matsumoto et al. (2011) reported research on personalized peptide vaccine (PPV) treatment for advanced urothelial cancer (UC) patients who failed to respond to methotrexate, vinblastine, adriamycin, and cisplatin (MVAC). Ten patients who were refractory or metastatic were treated with PPV 12 times weekly. Eight of them showed increased CTL immune response and anti-peptide IgG titers. Meanwhile, they also concluded that the FSP vaccines were safe, well tolerated and caused no serious adverse reactions in patients.

FSPs are potential sources of immunologically relevant antigens in cancers and are able to activate specific anti-tumour immune responses mediated by CTLs, which have important application value for targeted and precise treatment of carcinomas. Therefore, FSPs are expected to become targets of immunotherapy.

Importantly, MSI-related FSP antigens are caused by functional gene mutations. There is no evidence that an obvious difference exists in cMSI pattern between sporadic and hereditary MSI cancers, so FSP neoantigens between the two kinds of MSI cancers are shared. Therefore, due to the crucial functional significance of the underlying gene mutations in the tumourigenesis, FSP neoantigens may fulfil an indispensable prerequisite for the production of effective prophylactic vaccines that not are only applied to sporadic MSI cancers in an adjuvant setting but may also be relevant for hereditary MSI carcinomas. Furthermore, for precancerous lesions exhibiting cMSI or people who are cMS mutation carriers, prophylactic FSP vaccines will activate specific anti-tumour immune responses to eradicate underlying lesions or mutant cells with the goal of achieving an early prophylactic response.

## Conclusions

The occurrence of tumour is the result of multiple steps and multiple genes involved with the host genetic background and environmental factors. Understanding these factors has important clinical significance for tumourigenesis, progression, prognosis, and response to chemotherapy to explore MSI and FSPs, which are helpful for early screens of high-risk patients at the molecular level and to optimize treatment strategies. In the era of individualized and precise treatment, strategies based on FSP vaccines are projected to become a novel targeted therapy modality for malignancies.
